# A modified Delphi consensus on fusion (laparoscopic and robotic) approaches to colorectal surgery in Asia–Pacific

**DOI:** 10.1007/s00535-026-02445-x

**Published:** 2026-05-16

**Authors:** Jun Watanabe, Yoshinori Kagawa, Takashi Nonaka, Hyejin Kim, Siripong Cheewatanakornkul, Peter Chien-Chih Chen, William Tzu-Liang Chen, Ashwin deSouza, Atsushi Hamabe, Hiroyasu Kagawa, Yoon Suk Lee, Simon Siu-Man Ng, Andrew R. L. Stevenson, Nan Zun Teo, Mamoru Uemura, Kar Yong Wong, James Chi-Yong Ngu

**Affiliations:** 1 Department of Colorectal Surgery, Kansai Medical University, 2 Chome-5-1 Shinmachi, Hirakata, Osaka 573-1191 Japan; 2 Department of Gastroenterological Surgery, Osaka International Cancer Institute, Osaka, Japan; 3 Department of Surgical Oncology, Graduate School of Biomedical Sciences, Nagasaki University, Nagasaki, Japan; 4 Colorectal Cancer Center, Kyungpook National University Medical Chilgok Hospital, Daegu, South Korea; 5 Department of Surgery, Minimally Invasive Surgery Unit, Prince of Songkla University, Hat Yai, Thailand; 6 Department of Surgery, Sun Yat-Sen Cancer Center, Koo Foundation, Taipei, Taiwan; 7 College of Medicine, National Yang-Ming University, Taipei, Taiwan; 8 Department of Colorectal Surgery, China Medical University Hospital, China Medical University, Taichung, Taiwan; 9 Department of Colorectal Surgery, China Medical University Hsinchu Hospital, China Medical University, Hsinchu, Taiwan; 10 Department of Surgery, Tata Memorial Centre and Homi Bhabha National Institute, Mumbai, India; 11 Department of Gastroenterological Surgery, Graduate School of Medicine, The University of Osaka, Osaka, Japan; 12 Department of Gastrointestinal Surgery, Institute of Science Tokyo, Tokyo, Japan; 13 Division of Colorectal Surgery, Department of Surgery Seoul St. Mary Hospital, College of Medicine, The Catholic University of Korea, Seoul, South Korea; 14 Department of Surgery, The Chinese University of Hong Kong, Hong Kong SAR, China; 15 Department of Surgery, Division of Colorectal Surgery, The Royal Brisbane and Women’s Hospital, Brisbane, QLD Australia; 16 Department of Surgery, Changi General Hospital, Singapore, Singapore; 17 Department of General Surgery, Tan Tock Seng Hospital, Singapore, Singapore

**Keywords:** Colorectal cancer, Robot-assisted surgery, Laparoscopic surgery, Fusion surgery, Consensus

## Abstract

**Background:**

Fusion surgery, where laparoscopic techniques are purposefully integrated into robotic surgery, is a novel approach that can be used in colorectal surgery. However, a standard definition and practical guidance are lacking. Therefore, this study aimed to generate consensus to standardise the definition of fusion surgery and consequently raise awareness of fusion surgical approaches and their appropriate use cases.

**Methods:**

We conducted a two-round modified Delphi study with 17 colorectal surgeons from Asia–Pacific (5 Steering Committee [SC]; 12 Expert Panellists [EP]). Statements were drafted by the SC based on clinical experience, published literature and comments from the EP. The EP rated these statements anonymously on a 5-point Likert scale. A virtual expert meeting was held between voting rounds to discuss the statements. Consensus was predefined as ≥ 75% “agree”/”strongly agree” or “disagree”/”strongly disagree”.

**Results:**

Ultimately, consensus was reached on 23 statements: “Defining Fusion Surgery in Colorectal Surgery” (*n* = 2); “When to Consider Fusion Surgery in Colorectal Surgery” (*n* = 10; 5 statements each on “Device Factors” and “Surgeon Factors”); “Task- or Procedure-Specific Considerations in Colorectal Surgery” (*n* = 11).

**Conclusion:**

The Expert Panel were aligned on the definition of fusion surgery as a procedure where laparoscopic tools and techniques are electively combined with robotic surgery. Fusion surgery may achieve similar safety and effectiveness as fully robotic surgery, whilst potentially offering advantages such as improved ergonomics, shorter operating times and educational opportunities for bedside assistants. This consensus serves as a valuable guide containing considerations for surgeons to encourage the appropriate adoption of fusion approaches in colorectal surgery.

**Supplementary Information:**

The online version contains supplementary material available at 10.1007/s00535-026-02445-x.

## Introduction

Surgery is the mainstay of treatment for colorectal cancer, and is also used in other non-cancerous indications such inflammatory bowel disease [1–3]. The age-standardised global incidence rate of inflammatory bowel disease was 4.97 per 100,000 in 2019 [4], and the rate for colorectal cancer was approximately five times higher (26.7 per 100,000) [5]. Data from GLOBOCAN 2022 indicate that colorectal cancer is the third most common cancer globally, with over 1.9 million new cases annually [6]. Most of these cases were from Asia (~ 2.4 million cases), with China, Japan, South Korea, Malaysia and Singapore being amongst the Asian countries reporting the highest 5-year prevalence rates [7]. Colorectal cancer also ranks second highest in cancer-related mortality, accounting for approximately 900,000 annual deaths worldwide [6]. As most cases of colorectal cancer are treated surgically [8], colorectal cancer accounts for a large proportion of colorectal surgeries conducted globally.

Colorectal surgeries can be performed laparoscopically or with a conventional open approach; meta-analyses of randomised controlled trials in colorectal cancer indications have demonstrated superior safety (statistically significant reductions in blood loss, complications, hospital length of stay, mortality) and comparable clinical outcomes (long-term survival and recurrence) with laparoscopic approaches compared to open surgery [9, 10]. However, certain aspects of laparoscopic surgery may be challenging; for example, accessing the deep pelvic region may prove difficult due to limited visualisation and dexterity of laparoscopic instruments [11].

To address this, technological innovations such as robotic systems have been introduced. However, randomised controlled trials in rectal cancer have reported mixed findings when comparing robotic surgery to laparoscopic surgery. The ROLARR trial found no significant differences in overall rate of conversion and clinical endpoints (e.g. pathological outcomes, complications) between robotic and laparoscopic surgery [12], whilst the REAL trial demonstrated significantly lower conversion, complication and positive circumferential margin rates in favour of robotic surgery [13, 14]. Other drawbacks of robotic surgery, such as increased cost, longer operative time and limited tactile feedback, may also potentially hinder the use of the technology [11, 15, 16].

Leveraging the benefits of both surgical modes, combined approaches have emerged, where elements of laparoscopy are incorporated into robotic surgery [17, 18]. “Fusion” or “hybrid” surgery (thereafter referred to in this article as “fusion surgery”) are terms that have been coined to describe such procedures [18, 19], although a standardised definition has yet to be agreed upon. In order to establish best practices in fusion surgery, it is important for clinicians to consolidate their expertise and experiences to achieve a consensus on recommendations for fusion surgery [19]. This would promote greater awareness of fusion surgery as an option to improve surgical and patient outcomes.

Given the increasing adoption of robotic surgery in the Asia–Pacific (APAC) region, the objective of this study was to generate consensus amongst colorectal surgeons in APAC region to standardise the definition of fusion surgery, and consequently raise awareness of combined surgical approaches and their appropriate use cases. This study was conducted using a modified Delphi panel method [20]. Delphi panels offer a systematic, robust and reproducible methodology for developing guidance, via iterative rounds of voting on a series of consensus statements [21, 22].

## Methods

A modified Delphi method was used to support a group of expert colorectal surgeons in developing consensus statements on the use of fusion surgery, a combined approach comprising robotic and laparoscopic techniques, for colorectal indications in APAC. This was a two-round modified Delphi panel, with a group discussion meeting (hereby referred to as the “Virtual Expert Meeting”) between Round 1 and Round 2. No patient-level data were collected and no interventions were studied; the modified Delphi was based on the expert opinions of colorectal surgeons and published literature. Therefore, formal ethical approval was not required. This manuscript was written in accordance with the ACcurate COnsensus Reporting Document (ACCORD) guidelines [23].

### Expert selection and roles

This modified Delphi panel was carried out by a group of colorectal surgeons, which consisted of a Steering Committee and an Expert Panel. A study protocol, which was pre-specified but not registered online, outlined the selection criteria for the enrolment of experts. All participating experts are authors of this article. As this consensus exercise was being performed in a well-defined knowledge area, a smaller panel of experts was considered appropriate [20]. Previous publications have noted that a minimum of 12 voting panellists is generally considered sufficient to achieve consensus [24].

The Steering Committee consisted of 5 experts from countries in APAC where robotic surgery is advanced (Japan [*n* = 3]; Singapore [*n* = 1]; South Korea [*n* = 1]), who met the criteria listed in Table 1. The Steering Committee led the Delphi panel, which included advising on the scope of consensus exercise and overseeing the development of consensus statements; they did not participate in voting. The Steering Committee nominated and invited eligible colorectal surgeons to participate in the modified Delphi panel according to the Expert Panel criteria in Table 1. In addition to the listed selection criteria, the Expert Panel were prioritised for recruitment with the aim of geographical representativeness, to ensure a diversity of participants across APAC. The Expert Panel participated in the Delphi panel by anonymously voting on the statements developed by the Steering Committee, as well as providing open-ended feedback.

**Table 1:** Steering Committee and Expert Panel selection criteria

Steering Committee selection criteria	Expert Panel selection criteria
• Colorectal surgeons, licenced and actively practicing, preferably in a large or leading hospital (e.g. referral hospital, university hospital or public hospital) from Japan, Singapore or South Korea • Member of a relevant professional clinical organisation/society in respective regions • Published research focussed on colorectal surgery in the past 2 years • Extensive clinical experience with colorectal surgery • Able to commit to the time required to co-lead the Delphi panel • Able to provide constructive and critical feedback in English • Highly recognised in the field of colorectal surgery in Asia • A strong record of contributing to and leading discussions on colorectal surgery	• Colorectal surgeons, licenced and actively practicing, preferably in a large or leading hospital (e.g. referral hospital, university hospital or public hospital) in Asia • Member of a relevant professional clinical organisation/society in respective regions • Extensive clinical experience with colorectal surgery • Able to commit to the time required to participate in the Delphi panel • Able to provide constructive and critical feedback in English

The Steering Committee was provided an honorarium by the project sponsor for their time contributing to the modified Delphi panel. A third-party healthcare consultancy (Costello Medical), funded by the project sponsor, supported the modified Delphi panel by reviewing and consolidating evidence to inform and develop statements in collaboration with the Steering Committee, screening Expert Panel nominees against the selection criteria, and analysing anonymised responses to ensure blinded outputs. Costello Medical did not participate in voting. The project sponsor did not play a role in the development of statements, voting or the analysis of responses.

### Targeted searches

Targeted searches were conducted to identify relevant guidelines, consensus papers and clinical or economic studies published up to July 2024. Searches were conducted via PubMed and Google, as guidelines published in grey literature (e.g. medical society websites) may not be indexed in medical literature databases.

Search strategies used included search strings relating to fusion approaches (e.g. hybrid; combination of laparoscopic and robotic) and colorectal indications (e.g. colorectal cancer; colon; rectal). The first 50 hits of six search strings were screened. Amongst 51 potentially relevant publications, 18 high-priority publications reporting on a fusion approach (i.e. a combination of laparoscopic and robotic techniques) in colorectal indications were further reviewed and extracted.

The targeted searches informed the development of statements with the Steering Committee, alongside their clinical experience. The Expert Panel were able to review supporting evidence cited for each statement, where relevant, during each round of voting.

### Modified Delphi process

This modified Delphi panel was run under conditions of response anonymity, but not participation anonymity. This meant that voting was anonymised, and only Costello Medical were able to link responses to specific participants. However, Expert Panellists were aware of who was participating in each voting round of the Delphi panel.

The Steering Committee discussed and prioritised topics for the consensus statements based on their clinical experience, including at a face-to-face meeting held on 19th October 2024. Statements were grouped into three main topics: (1) “Defining Fusion Surgery in Colorectal Surgery”; (2) “When to Consider Fusion Surgery”; (3) “Task- or Procedure-Specific Considerations”. Statements under “When to Consider Fusion Surgery” were further categorised into subtopics: (1) “Device Factors”, (2) “Surgeon Factors” and (3) “Patient Factors”. Additionally, given that there are a variety of robotic platforms available with different characteristics that may impact practice, the Expert Panel were asked to indicate which robotic platforms they have used in the past and which robotic platform they most frequently use.

For each Delphi round, the statements and supporting references were distributed to the Expert Panel by e-mail and responses were collected via an online survey platform developed and managed by Costello Medical. No pilot survey was run for this modified Delphi panel. The Expert Panellists were asked to vote anonymously on a 5-point Likert scale (1—“Strongly Disagree” to 5—“Strongly Agree”), and were given the opportunity to provide feedback or comment on statements through open-text fields. Statements met consensus if ≥ 75% of respondents voted either “Agree”/“Strongly Agree” or “Disagree”/“Strongly Disagree”, a threshold which was pre-specified in the study protocol. Statements that reached consensus in Round 1 were not included for voting in the subsequent round.

Following the first round of voting, the Steering Committee chaired a Virtual Expert Meeting, which allowed the Expert Panel to review and discuss the statements that did not reach consensus after the first Delphi round. The Steering Committee presented the anonymised quantitative (proportion of respondents agreeing or disagreeing) and qualitative (free-text comments) results to the Expert Panel. The experts discussed the comments received for statements that did not reach consensus and provided suggestions to revise the statements. Minutes from the Virtual Expert Meeting were circulated to all Steering Committee and Expert Panel members after the meeting.

Statements that did not reach consensus during Round 1 were updated by the Steering Committee, based on feedback received from the Expert Panel, in preparation for Round 2 of the Delphi survey. During re-voting on revised statements during Round 2, the original statement and anonymised results from Round 1 were available for review. Any statements that did not reach consensus in Round 2 were removed from the final list of consensus statements.

## Results

### Round 1

**Fig. 1:**
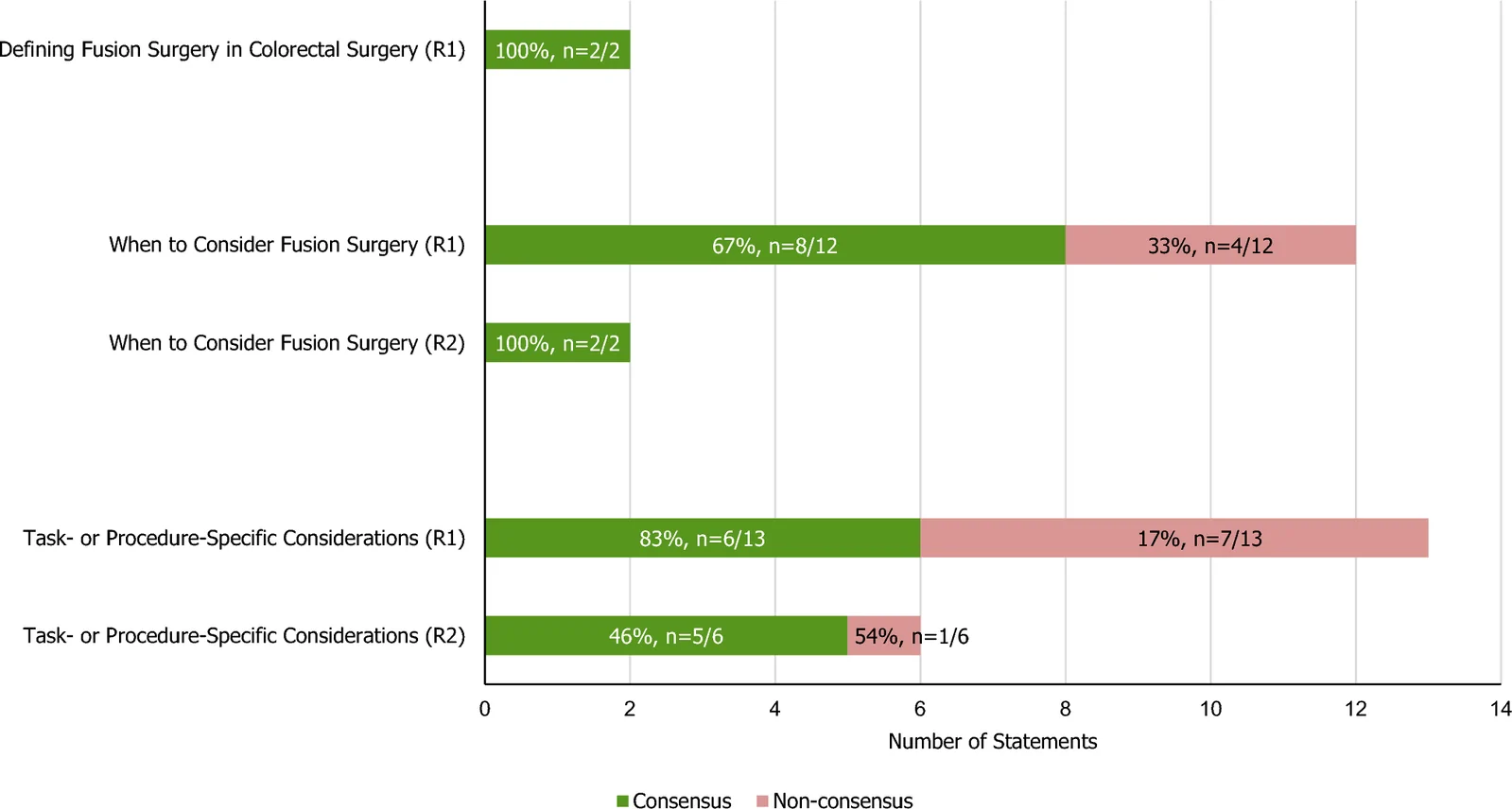
Number of statements achieving consensus or not achieving consensus by theme for Round 1 and Round 2

An Expert Panel of 12 colorectal surgeons was recruited via purposive sampling. The Expert Panellists were from Japan (*n* = 3), Singapore (*n* = 2), Taiwan (*n* = 2), Australia (*n* = 1), Hong Kong (*n* = 1), India (*n* = 1), South Korea (*n* = 1) and Thailand (*n* = 1). All Expert Panellists selected the Da Vinci Xi (Intuitive Surgical) as the most commonly used robotic platform in their practice. All Expert Panellists voted in the Round 1 survey, which was launched in December 2024 and closed in March 2025. Of the 27 statements initially proposed in Round 1, 16 statements reached consensus (59%) and 11 did not (41%). All statements under the “Defining Fusion Surgery in Colorectal Surgery” reached consensus (2/2, 100%). Comparatively, lower proportions of statements under “When to Consider Fusion Surgery” (8/12, 67%) and “Task- or Procedure-Specific Considerations” (6/13, 46%) reached consensus (Fig. 1).

### Virtual Expert Meeting

The 11 statements that did not reach consensus were discussed at the Virtual Expert Meeting (held on 21 st April 2025), attended by nine out of the 12 Expert Panellists and four out of the five Steering Committee members. Those unable to attend were invited to provide input over email. Based on the discussions during the Expert Meeting, statements that did not achieve consensus during Round 1 were rephrased, combined or removed for Round 2 voting. Of the 11 statements that did not reach consensus in Round 1, three non-consensus statements were removed from voting and the eight remaining non-consensus statements were revised into six statements for Round 2. Additionally, two new statements were proposed, resulting in a total of eight statements for voting in Round 2 (**Supplementary material 1**).

### Round 2

All Expert Panellists voted in the Round 2 survey, which was conducted from June 2025 until July 2025. Of the eight statements that were voted on in Round 2, seven statements reached consensus (88%) and one did not (12%). Both statements under the heading of “When to Consider Fusion Surgery” reached consensus (2/2, 100%). Of the six statements under the “Task- or Procedure-Specific Considerations” theme, five reached consensus (83%) (Fig. 1).

### Final consensus statements

Ultimately, across the two survey rounds, 23 statements reached consensus (Table 2). Two statements were on “Defining Fusion Surgery in Colorectal Surgery”. A total of ten statements under the “When to Consider Fusion Surgery in Colorectal Surgery” theme reached consensus, of which five statements were on “Device Factors” and another five statements were on “Surgeon Factors”. Eleven consensus statements were about “Task- or Procedure-Specific Considerations in Colorectal Surgery”. Full details on the evolution of the modified Delphi statements between rounds are available in **Supplementary material 1**.

**Table 2:** Final list of consensus statements

Statement	Consensus %
Defining fusion surgery in colorectal surgery	
1. Fusion or hybrid surgery is defined as a procedure combining conventional and robot-assisted laparoscopy, in which the surgeon electively chooses to either complete key components of the procedure using both techniques, and/or perform key tasks using instruments from both platforms	92% *Strongly agree: 58%* *Agree: 34%*
2. Fusion surgery does not include unplanned conversions from a robotic to a laparoscopic approach	92% *Strongly agree: 83%* *Agree: 8%*
When to consider fusion surgery in colorectal surgery—device factors	
3. There may be instances when a robotic platform lacks an equivalent feature or capability that exists in conventional laparoscopic equipment (e.g. clips, staplers, energy devices). In these situations, fusion surgery could be beneficial	92% *Strongly agree: 33%* *Agree: 59%*
4. Robotic platforms that allow sufficient access to the surgical field by the bedside assistant can be more suitable for fusion surgery (e.g. the robot has a slimmer profile or a suitable docking position)	92% *Strongly agree: 58%* *Agree: 34%*
5. The port configurations required by the robotic platform can affect the feasibility and ergonomics of fusion surgery	92% *Strongly agree: 42%* *Agree: 50%*
6. Fusion surgery using conventional laparoscopic devices for certain components of the procedure may be cost-saving compared to fully robotic surgery	75% *Strongly agree: 58%* *Agree: 17%*
7. Safety may likely be similar between fusion surgery and fully robotic surgery	75% *Strongly agree: 25%* *Agree: 50%*
When to consider fusion surgery in colorectal surgery—surgeon factors	
8. A competent bedside assistant is required for successful fusion surgery	100% *Strongly agree: 75%* *Agree: 25%*
9. In fusion surgery, there may be a greater reliance on the bedside assistant and therefore the need for better collaboration and coordination amongst the surgical team	83% *Strongly agree: 42%* *Agree: 41%*
10. As fusion surgery allows for greater participation by bedside assistants, it can allow them to feel more engaged and motivated	75% *Strongly agree: 17%* *Agree: 58%*
11. Fusion surgery can provide bedside assistants with opportunities for laparoscopic training	83% *Strongly agree: 25%* *Agree: 58%*
12. Performing part of a procedure laparoscopically in fusion surgery can potentially help to shorten the learning curve for robotic surgery for a new robotic surgeon	75% *Strongly agree: 17%* *Agree: 58%*
Task- or procedure-specific considerations in colorectal surgery	
13. For colectomies, fusion surgery with conventional laparoscopic staplers can achieve equivalent safety to robotic staplers	75% *Strongly agree: 42%* *Agree: 33%*
14. For rectal transection, fusion surgery with conventional laparoscopic staplers can achieve equivalent safety to robotic staplers	75% *Strongly agree: 25%* *Agree: 50%*
15. The decision to use a conventional laparoscopic or robotic stapler may be influenced by the ergonomics of using the device	92% *Strongly agree: 25%* *Agree: 67%*
16. When operating in areas that are more accessible (e.g. colon or high rectal transection), the ergonomics associated with using a conventional laparoscopic stapler can be comparable to that of a robotic stapler	83% *Strongly agree: 42%* *Agree: 42%*
17. When operating in areas that are less accessible (e.g. low rectal stapling within the pelvis), fusion surgery using a conventional laparoscopic stapler may be more challenging, and the use of a robotic stapler can have ergonomic advantages	92% *Strongly agree: 42%* *Agree: 50%*
18. In fusion surgery, if a bedside assistant operates the conventional laparoscopic stapler, the lead surgeon operating the robot can maintain active control of both robotic arms for tissue manipulation and retraction	92% *Strongly agree: 58%* *Agree: 34%*
19. The time required for stapling can be affected by many factors and may not solely depend on the choice of conventional laparoscopic or robotic staplers	92% *Strongly agree: 42%* *Agree: 50%*
20. For surgical fields involving multiple quadrants, fusion surgery may increase efficiency and shorten operating time	75% *Strongly agree: 42%* *Agree: 33%*
21. For oncological colorectal resections, the ability to achieve adequate resection margins can be similar between fusion surgery and fully robotic surgery	75% *Strongly agree: 33%* *Agree: 42%*
22. The addition of a laparoscopic advanced energy device used by a bedside assistant in fusion surgery can be complementary to the benefits of robotic surgery	75% *Strongly agree: 8%* *Agree: 67%*
23. When laparoscopic advanced energy devices are operated by the bedside assistant as part of fusion surgery, the robotic surgeon can maintain control and use of all robotic arms	92% *Strongly agree: 33%* *Agree: 59%*

There were four statements that did not reach consensus (Table 3). Three statements were under the theme of “When to Consider Fusion Surgery in Colorectal Surgery”. One statement on patient-related factors described the consideration of taking a fusion approach when there are space constraints such as a small abdominal cavity. Another statement was on surgeon-related factors, describing how participation as a bedside assistant in fusion surgery can provide training opportunities for robotic surgery. Whilst no Expert Panellist strongly disagreed with these two statements, approximately a third of experts voted “neutral” or “disagree”. Separately, one expert voted “strongly disagree” to a statement discussing a gradual reduction of reliance on the bedside assistant in fusion surgery as a robotic surgeon grows more proficient. Four other experts voted “neutral” for this non-consensus statement. The final non-consensus statement was a task-specific consideration regarding the potential time-saving aspect of laparoscopic clip applicators in colorectal surgery compared to robotic clip applicators. Whilst no Expert Panellist strongly disagreed, a third of experts voted “neutral” and one expert voted “disagree”.

**Table 3:** List of non-consensus statements

Statement	Consensus %
When to consider fusion surgery in colorectal surgery—patient factors	
Fusion surgery may be preferred when space constraints are present (e.g. small abdominal cavity) given that the external components of current robotic systems generally occupy a larger footprint than conventional laparoscopic instruments, thereby limiting the ability to adopt a fully robotic approach	67% *Strongly agree: 17%* *Agree: 50%* *Neutral: 17%* *Disagree: 16%* *Strongly disagree: 0%*
When to consider fusion surgery in colorectal surgery—surgeon factors	
Participating as a bedside assistant in fusion surgery can provide training opportunities for surgeons who are planning to perform robotic surgery	58% *Strongly agree: 33%* *Agree: 25%* *Neutral: 34%* *Disagree: 8%* *Strongly disagree: 0%*
When fusion surgery is used as part of the introduction/training process for new robotic surgeons, dependence on the bedside assistant can be gradually reduced as the surgeon-in-training becomes more proficient with operating the robot	58% *Strongly agree: 25%* *Agree: 33%* *Neutral: 34%* *Disagree: 0%* *Strongly disagree: 8%*
Task- or procedure-specific considerations in colorectal surgery	
The use of laparoscopic clips during fusion surgery may help to shorten operation time, compared with fully robotic surgery, by saving on the time required to undock and redock the robotic clip applicator	58% *Strongly agree: 17%* *Agree: 42%* *Neutral: 33%* *Disagree: 8%* *Strongly disagree: 0%*

## Discussion

This modified Delphi panel provides consensus statements on the definition of fusion surgery and considerations for its practical application in colorectal indications. Although all participating experts reported the Da Vinci Xi as their most commonly used platform, the consensus statements are intended to reflect general principles of fusion surgery rather than platform-specific recommendations. These principles are expected to be broadly applicable across different robotic systems, although platform-specific characteristics may influence their practical implementation.

The experts were aligned on the definition of fusion surgery as a procedure electively combining conventional and robot-assisted laparoscopic techniques to complete key components or tasks of the procedure using both techniques and/or instruments. In this context, “key components” can be interpreted to refer to critical procedural steps that directly influence surgical outcomes, such as dissection, vascular control, transection and anastomosis. In contrast, basic assisting manoeuvres (e.g. suction or passive retraction) are not included unless they involve active execution of a defined surgical task using laparoscopic instruments. Unplanned conversions (from fully robotic procedures to laparoscopic approaches) were excluded from the definition of fusion surgery. Additionally, the experts were largely aligned on most surgeon and device factors when considering when to use fusion surgery, as well as task- or procedure-specific considerations for fusion surgery in colorectal indications. However, there were four statements that did not reach consensus.

One statement on the consideration of patient-related factors for fusion surgery did not reach consensus in Round 1. This non-consensus statement described incorporating laparoscopic instruments into robotic surgery in patients with small abdominal cavities to potentially achieve certain steps that may not be possible to accomplish via a fully robotic approach (Table 3). The statement referenced a published narrative review which describes how smaller working spaces in areas such as the pelvis can result in significant robotic arm clashing, making robotic surgery challenging [25]. However, whilst robotic platforms have evolved to become more compact, the Expert Panellists noted that the same limitations in space would exist as long as the robot is deployed, either as a standalone platform or as part of a fusion procedure. A possible scenario where the robotic platform is undocked mid-procedure was discussed by the experts; for example, performing the dissection in a total or proctocolectomy using the robotic platform and then completing the rest of the procedure laparoscopically after undocking the robot. To our knowledge, there have been no publications explicitly studying the impact of such a practice on space constraints. Therefore, this statement was removed from voting in Round 2.

Nevertheless, there was consensus amongst the Expert Panel on how smaller robotic platforms that allow for sufficient bedside access to the surgical field can be more suitable for fusion surgery (Statement 4; Table 2). This statement was based on the clinical experience of the Steering Committee, and 92% of Expert Panellists voted in agreement. Whilst no member of the Expert Panel disagreed, one voted neutral; they noted that the need for significant laparoscopic assistance whilst the robotic system is docked could be considered a limitation of robotic surgery, particularly for robotic platforms which occupy a large bedside space.

Additionally, two statements on surgeon-related factors for consideration of fusion surgery did not reach consensus, both of which were based on clinical experience/opinions of the Steering Committee, rather than published evidence. One non-consensus statement described how participating as a bedside assistant in fusion surgery can provide learning opportunities for robotic training (Table 3). The Steering Committee intended to describe how an improved understanding of the robotic platform can be acquired through experience at the bedside [26, 27]. Published literature has reported that participating as a bedside assistant can help the surgeon-in-training visualise how the robotic arms interact during surgery [26]; this increased awareness may allow them to troubleshoot external arm collisions, as well as provide the necessary guidance to their bedside assistant in the future [26]. However, there were concerns amongst some members of the Expert Panel that the mainstay of robotic training involves actual control of the robotic instruments from the Surgeon Console. As such, this statement was removed from Round 2. Instead, the Expert Panel came to a consensus (83%) on a statement describing how participation as a bedside assistant can provide training opportunities for laparoscopic techniques, rather than robotic techniques (Statement 11; Table 2). This is aligned with previously published report describing how participating as a bedside assistant in fusion surgery can provide training for those who may not be sufficiently familiar with conventional laparoscopic devices [28].

Another non-consensus statement on bedside assistants described the reduced dependence on a beside assistant as the robotic surgeon becomes more proficient (Table 3), which the Steering Committee had intended to describe how dependence on a bedside assistant can be reduced as the learning curve of the robotic surgeon plateaus. The Expert Panel discussed that certain roles played by the bedside assistant, for example suctioning of the operative field or the application of haemostatic clips, might reflect the method in which the robotic surgeon chooses to conduct the surgery and do not necessarily reflect the proficiency of the surgeon. This statement was, therefore, removed from voting in Round 2.

Further emphasising the experts’ opinions that reliance on a bedside assistant can be necessary and useful, a statement describing how a surgeon can retain active control of the robotic arms when the bedside assistant operates the laparoscopic stapler reached consensus in Round 1 (Statement 18; Table 2). Whilst 92% agreed with this statement, the Expert Panel emphasised that the bedside assistant should be skilled and competent with laparoscopic tools in fusion surgery. Therefore, a statement highlighting the need for a competent bedside assistant was included for voting in Round 2; the Expert Panellists unanimously agreed that a competent bedside assistant is required for successful fusion surgery (Statement 8; Table 2). In alignment with the Expert Panellists’ opinions, a published consensus guideline on the skills required by a bedside assistant in robotic surgery noted that a collaborative approach with a bedside assistant is common, and that establishing necessary skills for all members involved in robot-assisted surgery is essential [29].

The last non-consensus statement was a task-specific consideration describing the potential time-saving aspect of laparoscopic clips compared to robotic clips (Table 3). The Steering Committee hypothesised that the time taken to undock the robotic clip applicator, reload it, and then to redock it would be more than that involved during the use of a laparoscopic equivalent. However, feedback from the Expert Panel concluded that certain scenarios may result in the use of laparoscopic clips being more time-consuming, such as instances when the ergonomics of the laparoscopic clip applicator are suboptimal. The experts ultimately agreed that the statement could have been an over-generalisation of this specific task.

Whilst the potential time-saving aspect of laparoscopic clip applicators in fusion surgery did not reach consensus, another more general statement on the efficiency of fusion surgery did (Statement 20; Table 2). The experts agreed that for operations involving multiple quadrants, combining laparoscopic and robotic techniques could potentially shorten operating time. Time-savings in surgery can contribute to cost-savings by reducing operating room utilisation time [30]. Indeed, in this Delphi survey, one statement regarding the overall potential of fusion surgery to be cost-saving compared to fully robotic surgery achieved consensus (Statement 6; Table 2). Achieving consensus on specific cost-related benefits of fusion surgery across experts from different regions may be challenging due to regional- and country-level differences in healthcare costing (e.g. prices of consumables). For example, one expert noted that most institutions would charge per fire of a robotic Hem-o-lok clip, and that the reprocessing and cleaning of clip applicators, if more than one is used in an operation, may lead to additional costs.

Furthermore, geographic differences in healthcare funding and reimbursement status with respect to the use of robotic platforms may also impact the overall adoption of fusion surgery. A Japanese database study observed that after the approval of the da Vinci robotic platform in 2018 for gastrointestinal surgeries under the Japanese National Health Insurance system, the number of monthly robotic surgeries nearly doubled [31]. Currently, robotic surgery is reimbursed in Japan for all patients with rectal cancer (since 2018) and colon cancer (since 2022), as well as in Australia for both benign and malignant indications (since 2019). In Thailand, robotic surgery is currently only reimbursed for rectal cancer surgery, but not for colon cancer surgery. Similarly, reimbursement for robotic surgery is limited in Hong Kong, where costs of robotic operations in public hospitals are reimbursed for total mesorectal excision only. However, robotic surgery is not currently reimbursed for any colorectal indications in other APAC regions, such as Singapore and Korea. As such, fusion surgery might be a more appealing approach in such economies, assuming subsidies are accorded to the laparoscopic equipment used.

Overall, there is consensus on the overall potential of fusion surgery to be cost-saving in appropriate contexts. Currently, there is an ongoing randomised controlled trial in Japan to assess the benefits of fusion surgery in colon cancer (jRCTs052240215); this trial will assess the differences in operating time between fusion surgery and fully robotic surgery as the primary endpoint [32]. Secondary endpoints for this trial include safety outcomes as well as costs of surgery [32]. Data from this trial, when available, may be able to further support or contextualise the cost-savings that may be possible with fusion surgery.

### Strengths and limitations

To our knowledge, this study is the first consensus generation activity to be undertaken on fusion surgery for colorectal indications. The strength of the Delphi method lies in the use of a structured approach to elicit anonymised expert views on a topic of debate, allowing the assessment of the extent of agreement and to resolve disagreements on a topic, through an iterative process with controlled feedback. The Delphi approach was modified with an Expert Meeting between Round 1 and Round 2, allowing discussion on non-consensus statements, whilst preserving anonymity of participant voting.

However, this study is limited by a number of factors. A pragmatic approach was taken in the conduct of targeted searches for the development of statements, aiming to gain an understanding of the current treatment landscape and practice guidelines, rather than a systematic review aiming to identifying all available evidence. Therefore, levels of evidence for the literature identified in our pragmatic searches were not assessed. Additionally, the modified Delphi method was limited to two rounds of voting, which limited opportunity for additional rounds to further investigate areas of non-consensus. Additionally, the consensus statements were developed in English, and all voting and discussions were also conducted in English, as this was a common language between experts from multilingual regions. This could have introduced some language bias, as experts who may be less comfortable with the English language may not have participated and thus could not contribute their insights. Nevertheless, consensus was able to be generated on nearly all statements within the two voting rounds conducted.

The current consensus reflects the experiences of 17 colorectal surgeons in the APAC region. As the participating surgeons are not evenly distributed across all countries within the APAC region, it cannot be ruled out that those practising in other countries or regions not represented by this study may hold different views; thus, there may be limitations to the generalisability of the findings. For example, as we were unable to recruit participants from mainland China despite targeted invitations, views and clinical expertise from this geography may not be represented. Finally, given the limited data published on fusion surgery, the consensus statements largely draw on clinical experience, rather than empirical data. Further updates should be considered to incorporate new evidence and evolving techniques in fusion surgery.

## Conclusion

A panel of colorectal surgeons from APAC were largely aligned on the definition of fusion surgery as a procedure where the surgeon electively combines laparoscopic tools and techniques with that of robotic surgery. Additionally, colorectal surgeons from APAC were aligned on various considerations for fusion surgery, including appropriate situations where surgeons should consider fusion approaches, as well as task- or procedure-specific considerations to improve outcomes. Incorporating laparoscopic tools and techniques into robotic surgery for colorectal indications is thought to achieve similar safety and effectiveness as fully robotic surgery, whilst potentially offering advantages such as improved ergonomics, shorter operating times and educational opportunities for bedside assistants.

However, consensus was not reached on some educational opportunities and resource-saving benefits of fusion surgery, perhaps due to institutional and regional differences in surgeon training and reimbursement systems. Whilst these consensus statements may be particularly useful for colorectal surgeons within APAC, the guidelines and principles for conducting fusion surgery may also be applicable on an international scale.

## Supplementary Information

Below is the link to the electronic supplementary material.

Supplementary file1 (DOCX 24 KB)

## Abbreviations

ACCORD: ACcurate COnsensus Reporting Document
APAC: Asia-Pacific
EP: Expert Panel
SC: Steering Committee

## References

[1] Chiorean EG, Nandakumar G, Fadelu T, et al. Treatment of patients with late-stage colorectal cancer: ASCO resource-stratified guideline. JCO Glob Oncol. 2020;6:414–38. 32150483 10.1200/JGO.19.00367 PMC7124947

[2] Costas-Chavarri A, Nandakumar G, Temin S, et al. Treatment of patients with early-stage colorectal cancer: ASCO resource-stratified guideline. J Glob Oncol. 2019;5:1-19 10.1200/JGO.18.00214 PMC6426503 30802158

[3] M’Koma AE. Inflammatory bowel disease: clinical diagnosis and surgical treatment-overview. Medicina B Aires. 2022. 10.3390/medicina58050567. 10.3390/medicina58050567 PMC9144337 35629984

[4] Zhou J-L, Bao J-C, Liao X-Y, et al. Trends and projections of inflammatory bowel disease at the global, regional and national levels, 1990–2050: a bayesian age-period-cohort modeling study. BMC Public Health. 2023;23(1):2507. 38097968 10.1186/s12889-023-17431-8 PMC10722679

[5] Sharma R, Abbasi-Kangevari M, Abd-Rabu R, et al. Global, regional, and national burden of colorectal cancer and its risk factors, 1990–2019: a systematic analysis for the Global Burden of Disease Study 2019. Lancet Gastroenterol Hepatol. 2022;7(7):627–47. 35397795 10.1016/S2468-1253(22)00044-9 PMC9192760

[6] Bray F, Laversanne M, Sung H, et al. Global cancer statistics 2022: GLOBOCAN estimates of incidence and mortality worldwide for 36 cancers in 185 countries. CA Cancer J Clin. 2024;74(3):229–63. 38572751 10.3322/caac.21834

[7] Wong MC, Ding H, Wang J, et al. Prevalence and risk factors of colorectal cancer in Asia. Intest Res. 2019;17(3):317–29. 31085968 10.5217/ir.2019.00021 PMC6667372

[8] Benitez Majano S, Di Girolamo C, Rachet B, et al. Surgical treatment and survival from colorectal cancer in Denmark, England, Norway, and Sweden: a population-based study. Lancet Oncol. 2019;20(1):74–87. 30545752 10.1016/S1470-2045(18)30646-6 PMC6318222

[9] Wang C-L, Qu G, Xu H-W. The short- and long-term outcomes of laparoscopic versus open surgery for colorectal cancer: a meta-analysis. Int J Colorectal Dis. 2014;29(3):309–20. 24445673 10.1007/s00384-013-1827-1

[10] Song XJ, Liu ZL, Zeng R, et al. A meta-analysis of laparoscopic surgery versus conventional open surgery in the treatment of colorectal cancer. Medicine (Baltimore). 2019;98(17):e15347. 31027112 10.1097/MD.0000000000015347 PMC6831213

[11] Vilsan J, Maddineni SA, Ahsan N, et al. Open, laparoscopic, and robotic approaches to treat colorectal cancer: a comprehensive review of literature. Cureus. 2023;15(5):e38956. 37313091 10.7759/cureus.38956 PMC10259746

[12] Jayne D, Pigazzi A, Marshall H, et al. Effect of robotic-assisted vs conventional laparoscopic surgery on risk of conversion to open laparotomy among patients undergoing resection for rectal cancer: the ROLARR randomized clinical trial. JAMA. 2017;318(16):1569–80. 29067426 10.1001/jama.2017.7219 PMC5818805

[13] Feng Q, Yuan W, Li T, et al. Robotic versus laparoscopic surgery for middle and low rectal cancer (REAL): short-term outcomes of a multicentre randomised controlled trial. Lancet Gastroenterol Hepatol. 2022;7(11):991–1004. 36087608 10.1016/S2468-1253(22)00248-5

[14] Feng Q, Yuan W, Li T, et al. Robotic vs laparoscopic surgery for middle and low rectal cancer: the REAL randomized clinical trial. JAMA. 2025;334(2):136–48. 40455621 10.1001/jama.2025.8123 PMC12131176

[15] Prete FP, Pezzolla A, Prete F, et al. Robotic versus laparoscopic minimally invasive surgery for rectal cancer: a systematic review and meta-analysis of randomized controlled trials. Ann Surg. 2018;267(6):1034–46. 28984644 10.1097/SLA.0000000000002523

[16] Ravendran K, Abiola E, Balagumar K, et al. A review of robotic surgery in colorectal surgery. Cureus. 2023;15(4):e37337. 37182014 10.7759/cureus.37337 PMC10169093

[17] Gómez Ruiz M, Lainez Escribano M, Cagigas Fernández C, et al. Robotic surgery for colorectal cancer. Ann Gastroenterol Surg. 2020;4(6):646–51. 33319154 10.1002/ags3.12401 PMC7726686

[18] Kobayashi T, Yamada N, Igarashi Y, et al. Fusion surgery in robotic-assisted sigmoid and rectosigmoid colectomy: a value-optimized stapling technique: a video vignette. Colorectal Dis. 2025;27(6):e70135. 40462372 10.1111/codi.70135

[19] Lynch AC, Ngu J, Ng SSM, et al. Consensus-led recommendations defining practical principles of achieving optimal surgical outcomes in robotic colorectal surgery in the Asia-Pacific region. J Robot Surg. 2023;17(2):457–63. 35773553 10.1007/s11701-022-01439-0 PMC10076381

[20] Akins RB, Tolson H, Cole BR. Stability of response characteristics of a Delphi panel: application of bootstrap data expansion. BMC Med Res Methodol. 2005;5(1):37. 16321161 10.1186/1471-2288-5-37 PMC1318466

[21] Nasa P, Jain R, Juneja D. Delphi methodology in healthcare research: how to decide its appropriateness. World J Methodol. 2021;11(4):116–29. 34322364 10.5662/wjm.v11.i4.116 PMC8299905

[22] Stewart D, Gibson-Smith K, MacLure K, et al. A modified Delphi study to determine the level of consensus across the European Union on the structures, processes and desired outcomes of the management of polypharmacy in older people. PLoS ONE. 2017;12(11):e0188348. 29155870 10.1371/journal.pone.0188348 PMC5695766

[23] Gattrell WT, Logullo P, van Zuuren EJ, et al. ACCORD (ACcurate COnsensus Reporting Document): a reporting guideline for consensus methods in biomedicine developed via a modified Delphi. PLOS Med. 2024;21(1):e1004326. 38261576 10.1371/journal.pmed.1004326 PMC10805282

[24] Vogel C, Zwolinsky S, Griffiths C, et al. A Delphi study to build consensus on the definition and use of big data in obesity research. Int J Obes. 2019;43(12):2573–86. 10.1038/s41366-018-0313-9 PMC6892733 30655580

[25] Marks JH, Perez RE, Salem JF. Robotic transanal surgery for rectal cancer. Clin Colon Rectal Surg. 2021;34(5):317–24. 34512199 10.1055/s-0041-1729864 PMC8428981

[26] Wong NW, Teo NZ, Ngu JC. Learning curve for robotic colorectal surgery. Cancers Basel. 2024. 10.3390/cancers16193420. 39410039 10.3390/cancers16193420 PMC11475096

[27] Kakiuchi Y, Kuroda S, Yoshida Y, et al. An effective surgical educational system in the era of robotic surgery: “Double-Surgeon Technique” in robotic gastrectomy for minimally invasive surgery. Langenbecks Arch Surg. 2024;410(1):20. 39731598 10.1007/s00423-024-03593-5 PMC11682005

[28] Yamamoto T, Fukuda M, Nakano R, et al. Transverse colectomy using the RoboLap cooperative technique for mid-transverse colon cancer – a video vignette. Colorectal Dis. 2024;26(11):2012–3. 39262032 10.1111/codi.17162

[29] Brian R, Murillo A, Gomes C, et al. Consensus guidelines on the bedside assistant skills required in robotic surgery. Surg Endosc. 2024;38(11):6406–12. 39227438 10.1007/s00464-024-11206-x PMC11525406

[30] Bertocchi E, Brunelli D, Squaranti T, et al. Cost saving analysis of an Enhanced Recovery After Surgery (ERAS) program for elective colorectal surgery in an ERAS qualified and training center. World J Surg. 2025;49(4):850–8. 40056394 10.1002/wjs.12548 PMC11994153

[31] Nishigori T, Ichihara N, Obama K, et al. Prevalence and safety of robotic surgery for gastrointestinal malignant tumors in Japan. Ann Gastroenterol Surg. 2022;6(6):746–52. 36338596 10.1002/ags3.12579 PMC9628217

[32] Watanabe J. Randomized controlled trial to evaluate the efficacy of hybrid robot-assisted surgery using a laparoscopic device in robotic-assisted colorectal cancer surgery (jRCTs052240215). https://jrct.mhlw.go.jp/en-latest-detail/jRCTs052240215. 2024.

