# Supplementary material for: A modified Delphi consensus on fusion (laparoscopic and robotic) approaches to colorectal surgery in Asia–Pacific
Source: J Gastroenterol. 2026 May 16;61(9):1284–94. doi: 10.1007/s00535-026-02445-x (PMC13506586; doi:10.1007/s00535-026-02445-x)
Supplement: Supplementary file 1 [file 535_2026_2445_MOESM1_ESM.docx]

SUPPLEMENTARY MATERIALS

Supplementary material 1. Evolution of modified Delphi statements

| No. | Round 1 Statement | Round 1 Outcome | Round 2 Statement | Round 2 Outcome |
| --- | --- | --- | --- | --- |
| Defining Fusion Surgery in Colorectal Surgery | | | | |
| 1 | Fusion or hybrid surgery is defined as a procedure combining conventional and robot-assisted laparoscopy, in which the surgeon electively chooses to either complete key components of the procedure using both techniques, and/or perform key tasks using instruments from both platforms. | 🗸  Consensus reached: 92%  *Strongly Agree: 58%*  *Agree: 34%* |  | |
| 2 | Fusion surgery does not include unplanned conversions from a robotic to a laparoscopic approach. | 🗸  Consensus reached: 92%  *Strongly Agree: 83%*  *Agree: 8%* |  | |
| When to Consider Fusion Surgery in Colorectal Surgery – Patient Factors | | | | |
| - | Fusion surgery may be preferred when space constraints are present (e.g. small abdominal cavity) given that the external components of current robotic systems generally occupy a larger footprint than conventional laparoscopic instruments, thereby limiting the ability to adopt a fully-robotic approach | ✘  Consensus not reached: 67%  *Strongly agree: 17%*  *Agree: 50% Neutral: 17%* *Disagree: 16% Strongly disagree: 0%* | *This statement was removed for Round 2* |  |
| When to Consider Fusion Surgery in Colorectal Surgery – Device Factors | | | | |
| 3 | There may be instances when a robotic platform lacks an equivalent feature or capability that exists in conventional laparoscopic equipment (e.g. clips, staplers, energy devices). In these situations, fusion surgery could be beneficial. | 🗸  Consensus reached: 92% *Strongly agree: 33%*  *Agree: 59%* |  | |
| 4 | Robotic platforms that allow sufficient access to the surgical field by the bedside assistant can be more suitable for fusion surgery (e.g. the robot has a slimmer profile or a suitable docking position). | 🗸  Consensus reached: 92% *Strongly agree: 58%*  *Agree: 34%* |  | |
| 5 | The port configurations required by the robotic platform can affect the feasibility and ergonomics of fusion surgery. | 🗸  Consensus reached: 92% *Strongly agree: 42%*  *Agree: 50%* |  | |
| 6 | Fusion surgery using conventional laparoscopic devices for certain components of the procedure may be cost-saving compared to fully-robotic surgery. | 🗸  Consensus reached: 75% *Strongly agree: 58%*  *Agree: 17%* |  | |
| 7 | Safety may likely be similar between fusion surgery and fully-robotic surgery. | 🗸  Consensus reached: 75% *Strongly agree: 25%*  *Agree: 50%* |  | |
| When to Consider Fusion Surgery in Colorectal Surgery – Surgeon Factors | | | | |
| 8 |  | | *Newly proposed statement:* A competent bedside assistant is required for successful fusion surgery. | 🗸  Consensus reached: 100% *Strongly agree: 75%*  *Agree: 25%* |
| 9 | In fusion surgery, there may be a greater reliance on the bedside assistant and therefore the need for better collaboration and co-ordination amongst the surgical team. | 🗸  Consensus reached 83% *Strongly agree: 42%*  *Agree: 41%* |  | |
| 10 | As fusion surgery allows for greater participation by bedside assistants, it can allow them to feel more engaged and motivated. | 🗸  Consensus reached: 75% *Strongly agree: 17%*  *Agree: 58%* |  | |
| 11 | Fusion surgery can provide bedside assistants with opportunities for laparoscopic training. | 🗸  Consensus reached: 83% *Strongly agree: 25%*  *Agree: 58%* |  | |
| - | Participating as a bedside assistant in fusion surgery can provide training opportunities for surgeons who are planning to perform robotic surgery. | ✘  Consensus not reached: 58%  *Strongly agree: 33%*  *Agree: 25% Neutral: 34%* *Disagree: 8% Strongly disagree: 0%* | *This statement was removed for Round 2* |  |
| 12 | Fusion surgery can help laparoscopic surgeons who are planning to perform robotic surgery shorten their learning curve as it allows the use of an existing skillset (conventional laparoscopy) to compensate for any deficiencies during robotic training. | ✘  Consensus not reached: 59%  *Strongly agree: 17%*  *Agree: 42% Neutral: 25%* *Disagree: 8% Strongly disagree: 8%* | Performing part of a procedure laparoscopically in fusion surgery can potentially help to shorten the learning curve for robotic surgery for a new robotic surgeon. | 🗸  Consensus reached: 75% *Strongly agree: 17%*  *Agree: 58%* |
| - | When fusion surgery is used as part of the introduction/training process for new robotic surgeons, dependence on the bedside assistant can be gradually reduced as the surgeon-in-training becomes more proficient with operating the robot. | ✘  Consensus not reached: 58%  *Strongly agree: 25%*  *Agree: 33% Neutral: 34%* *Disagree: 0% Strongly disagree: 8%* | *This statement was removed for Round 2* |  |
| Task- or Procedure-Specific Considerations in Colorectal Surgery | | | | |
| 13 | For colectomies, fusion surgery with conventional laparoscopic staplers can achieve equivalent safety to robotic staplers. | 🗸  Consensus reached: 75% *Strongly agree: 42%*  *Agree: 33%* |  | |
| 14 |  | | *Newly proposed statement:* For rectal transection, fusion surgery with conventional laparoscopic staplers can achieve equivalent safety to robotic staplers. | 🗸  Consensus reached: 75% *Strongly agree: 25%*  *Agree: 50%* |
| 15 | A surgeon’s choice between the use of a conventional laparoscopic or robotic stapler may be guided by the expected number of stapler firings. At the same time, the stapler used may also impact the number of stapler firings required. | ✘  Consensus not reached: 50%  *Strongly agree: 0%*  *Agree: 50% Neutral: 17%* *Disagree: 33% Strongly disagree: 0%* | The decision to use a conventional laparoscopic or robotic stapler may be influenced by the ergonomics of using the device. | 🗸  Consensus reached: 92% *Strongly agree: 25%*  *Agree: 67%* |
| 16 | When operating in areas that are more accessible (e.g. colon or high rectal transection), the ergonomics associated with using a conventional laparoscopic stapler can be comparable to that of a robotic stapler. | 🗸  Consensus reached: 83% *Strongly agree: 42%*  *Agree: 42%* |  | |
| 17 | When operating in areas that are less accessible (e.g. low rectal stapling within the pelvis), fusion surgery using a conventional laparoscopic stapler may be more challenging, and the use of a robotic stapler can have ergonomic advantages. | 🗸  Consensus reached: 92% *Strongly agree: 42%*  *Agree: 50%* |  | |
| 18 | In fusion surgery, if a bedside assistant operates the conventional laparoscopic stapler, the lead surgeon operating the robot can maintain active control of both robotic arms for tissue manipulation and retraction. | 🗸  Consensus reached: 92% *Strongly agree: 58%*  *Agree: 34%* |  | |
| 19 | For colon resection, the addition of a conventional laparoscopic stapler in fusion surgery may potentially shorten the time required for stapling. | ✘  Consensus not reached: 42%  *Strongly agree: 0%*  *Agree: 42% Neutral: 25%* *Disagree: 33% Strongly disagree: 0%* | *Statements reformulated:* The time required for stapling can be affected by many factors and may not solely depend on the choice of conventional laparoscopic or robotic staplers. | 🗸  Consensus reached: 92% *Strongly agree: 42%*  *Agree: 50%* |
|  | For high rectal resection, the addition of a conventional laparoscopic stapler in fusion surgery may potentially shorten the time required for stapling. | ✘  Consensus not reached: 25%  *Strongly agree: 0%*  *Agree: 25% Neutral: 33%* *Disagree: 42% Strongly disagree: 0%* |  |  |
|  | For low rectal resection, the addition of a conventional laparoscopic stapler in fusion surgery may not result in significant differences in time required for stapling. | ✘  Consensus not reached: 50%  *Strongly agree: 8%*  *Agree: 42% Neutral: 25%* *Disagree: 25% Strongly disagree: 0%* |  |  |
| 20 | For surgical fields involving multiple quadrants, fusion surgery may increase efficiency and shorten operating time. | 🗸  Consensus reached: 75% *Strongly agree: 42%*  *Agree: 33%* |  | |
| 21 | For oncological colorectal resections, the ability to achieve adequate resection margins can be similar between fusion surgery and fully-robotic surgery. | 🗸  Consensus reached: 75% *Strongly agree: 33%*  *Agree: 42%* |  | |
| 22 | For dissection and vessel sealing (e.g. of mesenteric tissue), the addition of a laparoscopic advanced energy device controlled by a bedside assistant in fusion surgery can be time-saving and improve the efficiency of robotic surgery. | ✘  Consensus not reached: 33%  *Strongly agree: 8%*  *Agree: 25% Neutral: 34%* *Disagree: 25% Strongly disagree: 8%* | The addition of a laparoscopic advanced energy device used by a bedside assistant in fusion surgery can be complementary to the benefits of robotic surgery. | 🗸  Consensus reached: 75% *Strongly agree: 8%*  *Agree: 67%* |
| 23 | When advanced energy devices are operated by the bedside assistant as part of fusion surgery, a robotic arm can be freed to perform other tasks. | ✘  Consensus not reached: 67%  *Strongly agree: 17%*  *Agree: 50% Neutral: 17%* *Disagree: 8% Strongly disagree: 8%* | When laparoscopic advanced energy devices are operated by the bedside assistant as part of fusion surgery, the robotic surgeon can maintain control and use of all robotic arms. | 🗸  Consensus reached: 92% *Strongly agree: 33%*  *Agree: 59%* |
| - | The use of laparoscopic clips during fusion surgery may help to shorten operation time, compared with fully-robotic surgery. | ✘  Consensus not reached: 58%  *Strongly agree: 0%*  *Agree: 58% Neutral: 34%* *Disagree: 8% Strongly disagree: 0%* | *Statement reformulated:* The use of laparoscopic clips during fusion surgery may help to shorten operation time, compared with fully-robotic surgery, by saving on the time required to undock and redock the robotic clip applicator. | ✘  Consensus not reached: 58%  *Strongly agree: 17%*  *Agree: 42% Neutral: 33%* *Disagree: 8% Strongly disagree: 0%* |
